# Chatbot breakthrough in the 2020s? An ethical reflection on the trend of automated consultations in health care

**DOI:** 10.1007/s11019-021-10049-w

**Published:** 2021-09-04

**Authors:** Jaana Parviainen, Juho Rantala

**Affiliations:** Faculty of Social Sciences, Tampere University, Tampere, Finland

**Keywords:** Chatbot, Health care, Expertise, Professional ethics, COVID-19

## Abstract

Many experts have emphasised that chatbots are not sufficiently mature to be able to technically diagnose patient conditions or replace the judgements of health professionals. The COVID-19 pandemic, however, has significantly increased the utilisation of health-oriented chatbots, for instance, as a conversational interface to answer questions, recommend care options, check symptoms and complete tasks such as booking appointments. In this paper, we take a proactive approach and consider how the emergence of task-oriented chatbots as partially automated consulting systems can influence clinical practices and expert–client relationships. We suggest the need for new approaches in professional ethics as the large-scale deployment of artificial intelligence may revolutionise professional decision-making and client–expert interaction in healthcare organisations. We argue that the implementation of chatbots amplifies the project of rationality and automation in clinical practice and alters traditional decision-making practices based on epistemic probability and prudence. This article contributes to the discussion on the ethical challenges posed by chatbots from the perspective of healthcare professional ethics.

## Introduction

The emergence of COVID-19 as a global pandemic has significantly advanced the development of telehealth and the utilisation of health-oriented chatbots in the diagnosis and treatment of coronavirus infection (AlgorithmWatch 2020; McGreevey et al. 2020). COVID-19 screening is considered an ideal application for chatbots because it is a well-structured process that involves asking patients a series of clearly defined questions and determining a risk score (Dennis et al. 2020). For instance, in California, the Occupational Health Services did not have the resources to begin performing thousands of round-the-clock symptom screenings at multiple clinical sites across the state (Judson et al. 2020). To limit face-to-face meetings in health care during the pandemic, chatbots have being used as a conversational interface to answer questions, recommend care options, check symptoms and complete tasks such as booking appointments. In addition, health chatbots have been deemed promising in terms of consulting patients in need of psychotherapy once COVID-19-related physical distancing measures have been lifted.

Many health professionals and experts have emphasised that chatbots are not sufficiently mature to be able to technically diagnose patient conditions or replace health professional assessments (Palanica et al. 2019). Before the outbreak of the COVID-19 pandemic, the development of digital services and the integration of artificial intelligence-(AI) driven solutions, including chatbots, into public health care was sluggish, although the EU, for instance, had showed its willingness to invest heavily in the adoption of AI technologies in the healthcare sector (Ministry of Economic Affairs and Employment 2017). Although some applications can provide assistance in terms of real-time information on prognosis and treatment effectiveness in some areas of health care, health experts have been concerned about patient safety (McGreevey et al. 2020). Recent studies have indicated that health organisations have intensified their collaboration with the technology industry to scale up digital solutions for health professionals, patients and their families and capitalise on existing social media and health applications to reach key stakeholders quickly, such as at-risk vulnerable groups (Atique et al. 2020; Tiirinki et al. 2020, p. 660). A pandemic can accelerate the digitalisation of health care, but not all consequences are necessarily predictable or positive from the perspectives of patients and professionals. For instance, if primary healthcare services are increasingly built on chatbots and other digital solutions, the tech industry will increasingly gain in health care and contribute to the ‘corporate privatization of public functions’ (Suarez-Villa 2012, p. 188).

In this article, we take a proactive approach to the potential chatbot breakthrough in health care in relation to the COVID-19 pandemic and consider how the emergence of task-oriented chatbots as partially automated consulting systems can influence clinical practices. Based on findings from recent empirical studies of health chatbots, we approach the topic from the perspective of professional ethics and consider professional–patient relations and the changing positions of these stakeholders on health and medical assessments. Drawing on Aristotle’s account of *phronesis*, several authors (Conroy et al. 2021; Kaldjian 2014; Montgomery 2006; Oakley and Cocking 2001; Pellegrino and Thomasma 1993; Toon 2014) have developed theoretical accounts of the nature of the practical wisdom needed in medicine to promote good judgement in morally complex situations. In these ethical discussions, technology use is frequently ignored, technically automated mechanical functions are prioritised over human initiatives, or tools are treated as neutral partners in facilitating human cognitive efforts. So far, there has been scant discussion on how digitalisation, including chatbots, transform medical practices, especially in the context of human capabilities in exercising practical wisdom (Bontemps-Hommen et al. 2019).

We focus on a single chatbot category used in the area of self-care or that precedes contact with a nurse or doctor. These chatbots are variously called dialog agents, conversational agents, interactive agents, virtual agents, virtual humans or virtual assistants (Abd-Alrazaq et al. 2020; Palanica et al. 2019). For instance, in the case of a digital health tool called Buoy or the chatbot platform Omaolo, users enter their symptoms and receive recommendations for care options. Both chatbots have algorithms that calculate input data and become increasingly smarter when people use the respective platforms. The increasing use of bots in health care—and AI in general—can be attributed to, for example, advances in machine learning (ML) and increases in text-based interaction (e.g. messaging, social media, etc.) (Nordheim et al. 2019, p. 5). Chatbots are based on combining algorithms and data through the use of ML techniques. Their function is thought to be the delivery of new information or a new perspective. However, in general, AI applications such as chatbots function as tools for ensuring that available information in the evidence base is properly considered.

Advocates for the use of chatbots in health care have argued that algorithm-driven systems can free overworked professionals (Topol 2019), reduce the risk of errors (Paredes 2018), provide predictive analysis based on historical and real-time data (Pryce et al. 2018) and increase efficiency in the public sector (Accenture Consulting 2018). They expect that algorithms can make more objective, robust and evidence-based clinical decisions (in terms of diagnosis, prognosis or treatment recommendations) compared to human healthcare providers (HCP) (Morley et al. 2019). Thus, chatbot platforms seek to automate some aspects of professional decision-making by systematising the traditional analytics of decision-making techniques (Snow 2019). In the long run, algorithmic solutions are expected to optimise the work tasks of medical doctors in terms of diagnostics and replace the routine tasks of nurses through online consultations and digital assistance. Nevertheless, many experts (e.g. Eubanks 2017; Gavaghan et al. 2019; Wachter 2015) have stated that automated systems are not sufficiently reliable to be left to operate independently and that there needs to be a consideration of those factors that are not readily automatable or situations in which a measure of discretion is, for whatever reason, desirable. In addition, the development of algorithmic systems for health services requires a great deal of human resources, for instance, experts of data analytics whose work also needs to be publicly funded. A complete system also requires a ‘back-up system’ or practices that imply increased costs and the emergence of new problems. The crucial question that policy-makers are faced with is what kind of health services can be automated and translated into machine readable form.

The design principles of most health technologies are based on the idea that technologies should mimic human decision-making capacity. These systems are computer programmes that are ‘programmed to try and mimic a human expert’s decision-making ability’ (Fischer and Lam 2016, p. 23). Thus, their function is to solve complex problems using reasoning methods such as the if-then-else format. In the early days, the problem of these systems was ‘the complexity of mapping out the data in’ the system (Fischer and Lam 2016, p. 23). Today, advanced AI technologies and various kinds of platforms that house big data (e.g. blockchains) are able to map out and compute in real time most complex data structures. In addition, especially in health care, these systems have been based on theoretical and practical models and methods developed in the field. For example, in the field of psychology, so-called ‘script theory’ provided a formal framework for knowledge (Fischer and Lam 2016). Thus, as a formal model that was already in use, it was relatively easy to turn it into algorithmic form. These expert systems were part of the automated decision-making (ADM) process, that is, a process completely devoid of human involvement, which makes final decisions on the basis of the data it receives (European Commission 2018, p. 20). Conversely, health consultation chatbots are partially automated proactive decision-making agents that guide the actions of healthcare personnel.

Our discussion proceeds in three sections. First, we introduce health chatbots and their historical background and clarify their technical capabilities to support the work of healthcare professionals. Second, we consider how the implementation of chatbots amplifies the project of rationality and automation in professional work as well as changes in decision-making based on epistemic probability. We then discuss ethical and social issues relating to health chatbots from the perspective of professional ethics by considering professional-patient relations and the changing position of these stakeholders on health and medical assessments. Finally, to ground our analysis, we employ the perspective of HCPs and list critical aspects and challenges relating to how chatbots may transform clinical capabilities and change patient-clinician relationships in clinical practices in the long run. We stress here that our intention is not to provide empirical evidence for or against chatbots in health care; it is to advance discussions of professional ethics in the context of novel technologies. The paper’s main contributions are theoretical and philosophical in nature.

## Health consultation chatbots

Following Pasquale (2020), we can divide the use of algorithmic systems, such as chatbots, into two strands. First, there are those that use ML ‘to derive new knowledge from large datasets, such as improving diagnostic accuracy from scans and other images’. Second, ‘there are user-facing applications […] which interact with people in real-time’, providing advice and ‘instructions based on probabilities which the tool can derive and improve over time’ (p. 55). The latter, that is, systems such as *chatbots*, seem to complement and sometimes even substitute HCP patient consultations (p. 55).

The most famous chatbots currently in use are Siri, Alexa, Google Assistant, Cordana and XiaoIce. Despite their popularity, they are not used in health care. Two of the most popular chatbots used in health care are the mental health assistant Woebot and Omaolo, which is used in Finland. From the emergence of the first chatbot, ELIZA, developed by Joseph Weizenbaum (1966), chatbots have been trying to ‘mimic human behaviour in a text-based conversation’ (Shum et al. 2018, p. 10; Abd-Alrazaq et al. 2020). Thus, their key feature is language and speech recognition, that is, natural language processing (NLP), which enables them to understand, to a certain extent, the language of the user (Gentner et al. 2020, p. 2).

As Lee et al. (2020, p. 339) have pointed out, chatbots ‘can be classified based on their purpose (i.e., assistant or conversation) and mode of communication (i.e., text or voice)’. Their purpose can be divided into two categories: task-oriented (or goal-oriented) and social (Galitsky 2019, p. 3). The task-oriented purpose usually has to do with being an ‘assistant’, while the social role is ‘conversational’ in nature. The former employs a question–answer style or step-by-step conversation. These chatbots depend on ‘supervised learning, reinforcement learning and an extensive domain-specific knowledge’ (p. 3). The latter, namely social chatbots, are not usually task-oriented, unless the ‘sociality’ itself is the task or goal. This might be the case especially in healthcare chatbots that strive to help with mental health.

In the healthcare field, in addition to the above-mentioned Woebot, there are numerous chatbots, such as Your.MD, HealthTap, Cancer Chatbot, VitaminBot, Babylon Health, Safedrugbot and Ada Health (Palanica et al. 2019). Most of them are simple, user-faced and task-oriented (similar to ELIZA). One example of a task-oriented chatbot is a medical chatbot called Omaolo developed by the Finnish Institute for Health and Welfare (THL), which is an online symptom assessment tool (e-questionnaire) (Atique et al. 2020, p. 2464; THL 2020). The chatbot is available in Finnish, Swedish and English, and it currently administers 17 separate symptom assessments. However, what makes Omaolo more than an automated questionnaire is ‘an electronic engine of medical knowledge, which operates according to evidence-based medical information […]’ and ‘combines the symptoms, measurement results and health information reported by the resident with research data and the official care guidelines’ (THL 2020, p. 10). Omaolo-related instructions underline that ‘answers are assessed mechanically with software built on the logical [and automatic] reasoning of’ the Finnish Medical Society Duodecim’s Evidence-Based Medicine Electronic Decision Support (EBMEDS) (THL 2020, p. 12; Duodecim 2020, p. 2). Omaolo has a variety of purposes. First, it can perform an assessment of a health problem or symptoms and, second, more general assessments of health and well-being. Third, it can perform an ‘assessment of a sickness or its risks’ and guide ‘the resident to receive treatment in services promoting health and well-being within Omaolo and in social and health services external to’ it (THL 2020, p. 14). Fourth, it offers quality-of-life surveys, oral health surveys and health coaching. In the aftermath of COVID-19, Omaolo was updated to include ‘Coronavirus symptoms checker’, a service that ‘gives guidance regarding exposure to and symptoms of COVID-19’ (Atique et al. 2020, p. 2464; Tiirinki et al. 2020). In September 2020, the THL released the mobile contact tracing app Koronavilkku,^1^ which can collaborate with Omaolo by sharing information and informing the app of positive test cases (THL 2020, p. 14).

The COVID-19 pandemic has arguably accelerated the development of digital health services within the Finnish healthcare system, and Omaolo ‘may have…potentially prevented unnecessary visits to healthcare facilities’ (Tiirinki et al. 2020, p. 660). A small survey (N = 11) conducted by nursing students (Pynnönen et al. 2020) on the HCP use of Omaolo concluded that those interviewed predicted an increase in the use of the bot as patients got used to the service. From an HCP perspective, Omaolo seemed to save work time (p. 24). Also, Judson et al. (2020, p. 1453) estimated that a partially similar chatbot, Conversa, which they designed, had ‘saved employees over 15 000 h of time waiting to be screened’ (from March to May 2020).

Nevertheless, what are the strengths of chatbot technologies in health care? In general, it is thought that healthcare chatbots ‘have the potential to provide patients with access to immediate medical information, recommend diagnoses at the first sign of illness, or connect patients with suitable health care providers (HCPs) across their community’ (Palanica et al. 2019). In addition, people seem to think that as bots are devoid (usually) of human features (e.g. gender or age), they are seen as more trustworthy and non-judgmental, and people are ‘more willing to disclose medical information to them’ (Palanica et al. 2019; Dennis et al. 2020; Judson et al. 2020). In the case of Omaolo, for example, it seems that it was used extensively for diagnosing conditions that were generally considered intimate, such as urinary tract infections and sexually transmitted diseases (STDs) (Pynnönen et al. 2020, p. 24). The presumed neutrality also helps with the fact that, as organizations such as the WHO have cautioned, ‘the COVID-19 outbreak has provoked social stigma and discriminatory behaviours against… those perceived to have been in contact with the virus’ (Dennis et al. 2020, p. 1730). Bots also do not get sick or tired, and they can be up and running 24 h per day. This relieving of pressure on contact centres is especially important in the present COVID-19 situation (Dennis et al. 2020, p. 1727), thus making chatbots cost-effective. However, one of the key elements for bots to be trustworthy—that is, the ability to function effectively with a patient—‘is that people believe that they have expertise’ (Nordheim et al. 2019). A survey on Omaolo (Pynnönen et al. 2020, p. 25) concluded that users were more likely to be in compliance with and more trustworthy about HCP decisions.

## Healthcare professionals and new decision-making conditions

With the increasing popularity of conversational agents in healthcare spaces involving the COVID-19 pandemic, medical experts (e.g. McGreevey et al. 2020) have become concerned about the consequences of these emerging technologies on clinical practices. It is unlikely that consultation chatbots, which are partially automated systems, can replace medical doctors in health care; however, they can drastically change the professional-patient relationship and decision-making processes in terms of diagnosis and treatment. One of the consequences can be the shift from operator to supervisor, that is, expert work becomes more about monitoring and surveillance than before (Zerilli et al. 2019). Complex algorithmic systems represent a growing resource of interactive, autonomous and often self-learning (in the ML sense) agency, potentially transforming cooperation between machines and professionals by emphasising the agency of machines (Morley et al. 2019). As a profound socio-digital change, partial ADM presumably transforms healthcare professionals’ work conditions, the skills required for jobs and, therefore, in the long run, the education system and re-arranges embodied and emotional relationships between professionals and customers. Thus, instead of only re-organising work, we are talking about *systemic change* (e.g. Simondon 2017), that is, change that pervades all parts of a system, taking into account the interrelationships and interdependencies among these parts.

The development—especially conceptual in nature—of ADM has one of its key moments in the aftermath of World War II, that is, the era of the Cold War. America and the Soviets were both keen (in their own ways) on find ways to automatise and streamline their societies (including decision-making). As Erickson et al. (2013, p. 21) pointed out, ‘operations research, game theory, strategic deterrence, linear programming, decision theory, and the experimental social sciences all seemed to be converging on similar problems with similar tools and standards for what constituted a satisfactory solution’. This was led by people from different fields of science, who were reconceptualising human reason ‘as rationality’ (p. 29), thus creating formal models of functions and processes of biological and artificial organisms, firms, organisations and even societies. In the field of medical practice, probability assessments has been a recurring theme. Mathematical or statistical probability in medical diagnosis has become one of the principal targets, with the consequence that AI is expected to improve diagnostics in the long run. Hacking (1975) has reminded us of the dual nature between statistical probability and epistemic probability. Statistical probability is concerned with ‘stochastic laws of chance processes’, while epistemic probability gauges ‘reasonable degrees of belief in propositions quite devoid of statistical background’ (p. 12). Epistemic probability concerns our possession of knowledge, or information, meaning how much support is given by all the available evidence.

When physicians observe a patient presenting with specific signs and symptoms, they assess the subjective probability of the diagnosis. Such probabilities have been called *diagnostic probabilities* (Wulff et al. 1986), a form of epistemic probability. In practice, however, clinicians make diagnoses in a more complex manner, which they are rarely able to analyse logically (Banerjee et al. 2009). Encountering the unexpected is an occupational hazard in clinical practice. Unlike artificial systems, experienced doctors recognise the fact that diagnoses and prognoses are always marked by varying degrees of uncertainty. They are aware that some diagnoses may turn out to be wrong or that some of their treatments may not lead to the cures expected. Thus, medical diagnosis and decision-making require ‘prudence’, that is, ‘a mode of reasoning about contingent matters in order to select the best course of action’ (Hariman 2003, p. 5).

Prudence or *prudentia* is Cicero’s Latin translation of the Greek *phronesis*, which is Aristotle’s (1926) formulation of practical wisdom in the *Nicomachean Ethics* (Hariman 2003, p. iv). According to Heinrichs (2007), Aristotle defined phronesis as the skill of dealing with probability, combining the ability to predict based on evidence and the ability to make decisions that produce the greatest probability of happiness. Phronesis, prudence or practical wisdom refers to the flexible, interpretive capacity that enables the physician to determine the best course of action when knowledge depends on circumstance (Montgomery 2006). Following Heinrichs’ (2007, p. 289) account of phronesis, we emphasise three constituent elements in medical decision-making: (1) showing off experience and knowledge, (2) bending the rules (i.e. adjusting to changing circumstances rather than following strict rules) and (3) taking the middle course (conciliatory view between contradictions and conflicts). Therefore, decision-making is a carefully articulated mode of reasoning, with its own specific standards, procedures and problems. Experienced doctors, as prudential actors, are capable of working in a challenging triad of messy practice, ethical standards and context-sensitive scientific knowledge. As Powell (2019, p. 2) put it, ‘what doctors often need is wisdom rather than intelligence, and we are a long way away from a science of artificial wisdom’.

Since the 1950s, there have been efforts aimed at building models and systematising physician decision-making. For example, in the field of psychology, the so-called framework of ‘script theory’ was ‘used to explain how a physician’s medical diagnostic knowledge is structured for diagnostic problem solving’ (Fischer and Lam 2016, p. 24). According to this theory, ‘the medical expert has an integrated network of prior knowledge that leads to an expected outcome’ (p. 24). As such models are formal (and have already been accepted and in use), it is relatively easy to turn them into algorithmic form. The rationality in the case of models and algorithms is instrumental, and one can say that an algorithm is ‘the conceptual embodiment of instrumental rationality within’ (Goffey 2008, p. 19) machines. Thus, algorithms are an actualisation of reason in the digital domain (e.g. Finn 2017; Golumbia 2009). The rationalisation of expert processes and knowledge go hand in hand. However, it is worth noting that formal models, such as game-theoretical models, do not completely describe reality or the phenomenon in question and its processes; they grasp only a slice of the phenomenon. This creates limitations regarding what ADM, partial ADM or chatbots can do.

Task-oriented chatbots follow these models of thought in a precise manner; their functions are easily derived from prior expert processes performed by humans. However, more conversational bots, for example, those that strive to help with mental illnesses and conditions, cannot be constructed—at least not easily—using these thought models. This requires the same kind of plasticity from conversations as that between human beings. The division of task-oriented and social chatbots requires additional elements to show the relation among users, experts (professionals) and chatbots. Most chatbot cases—at least task-oriented chatbots—seem to be user facing, that is, they are like a ‘gateway’ between the patient and the HCP. Only the data collected and delivered by chatbots are professional facing. Chatbots are trained (ML) with the help of health data that come directly from or are based on ‘both structured data sources such as EMR (Electronic Medical Records) as well as unstructured forms such as doctors’ notes, prescriptions, medical sensors, electronic monitors, mobile applications and research data bases’ (Harerimana et al. 2018, 65,663).

Through chatbots (and their technical functions), we can have only a very limited view of medical knowledge. The ‘rigid’ and formal systems of chatbots, even with the ML bend, are locked in certain a priori models of calculation. Expertise generally requires the intersubjective circulation of knowledge, that is, a pool of dynamic knowledge and intersubjective criticism of data, knowledge and processes (e.g. Prior 2003; Collins and Evans 2007). Therefore, AI technologies (e.g. chatbots) should not be evaluated on the same level as human beings. AI technologies can perform some narrow tasks or functions better than humans, and their calculation power is faster and memory more reliable. However, occasionally, these technologies are presented, more or less implicitly, as replacements of the human actor on a task, suggesting that they—or their abilities/capabilities—are identifiable with human beings (or their abilities/capabilities).

Furthermore, there are work-related and ethical standards in different fields, which have been developed through centuries or longer. For example, as Pasquale argued (2020, p. 57), in medical fields, science has made medicine and practices more reliable, and ‘medical boards developed standards to protect patients from quacks and charlatans’. Thus, one should be cautious when providing and marketing applications such as chatbots to patients. The application should be in line with up-to-date medical regulations, ethical codes and research data. Pasquale pointed to an Australian study of 82 mobile apps ‘marketed to those suffering from bipolar disorder’, only to find out that ‘the apps were, in general, not in line with practice guidelines or established self-management principles’ (p. 57).

One of the key elements of expertise and its recognition is that patients and others can trust the opinions and decisions offered by the expert/professional. However, in the case of chatbots, ‘the most important factor for explaining trust’ (Nordheim et al. 2019, p. 24) seems to be expertise. People can trust chatbots if they are seen as ‘experts’ (or as possessing expertise of some kind), while expertise itself requires maintaining this trust or trustworthiness. Chatbot users (patients) need to see and experience the bots as ‘providing answers reflecting knowledge, competence, and experience’ (p. 24)—all of which are important to trust. In practice, ‘chatbot expertise’ has to do with, for example, giving a correct answer (provision of accurate and relevant information). The importance of providing correct answers has been found in previous studies (Nordheim et al. 2019, p. 25), which have ‘identified the perceived ability of software agents as a strong predictor of trust’. Conversely, automation errors have a negative effect on trust—‘more so than do similar errors from human experts’ (p. 25). However, the details of experiencing chatbots and their expertise as trustworthy are a complex matter. As Nordheim et al. have pointed out, ‘the answers not only have to be correct, but they also need to adequately fulfil the users’ needs and expectations for a good answer’ (p. 25). Importantly, in addition to human-like answers, the perceived human-likeness of chatbots in general can be considered ‘as a likely predictor of users’ trust in chatbots’ (p. 25).

## Chatbots and the pressure on professional ethics

In the last decade, medical ethicists have attempted to outline principles and frameworks for the ethical deployment of emerging technologies, especially AI, in health care (Beil et al. 2019; Mittelstadt 2019; Rigby 2019). As conversational agents have gained popularity during the COVID-19 pandemic, medical experts have been required to respond more quickly to the legal and ethical aspects of chatbots. McGreevey et al. (2020) have addressed a number of ethical challenges that have arisen in relation to chatbots in health care, including patient safety; trust and transparency between all participants; content sources of the chatbots’ recommendations; cybersecurity threats; data use, privacy and integration and bias and health equity. To contribute to this discussion, we focus on clinical decision-making and client–expert interaction and review previous research findings on chatbots to respond to the question of what kinds of clinical tasks should or should not be augmented or automated by chatbots.

Dennis et al. (2020) examined ability, integrity and benevolence as potential factors driving trust in COVID-19 screening chatbots, subsequently influencing patients’ intentions to use chatbots and comply with their recommendations. They concluded that high-quality service provided by COVID-19 screening chatbots was critical but not sufficient for widespread adoption. The key was to emphasise the chatbot’s ability and assure users that it delivers the same quality of service as human agents (Dennis et al. 2020, p. 1727). Their results suggest that the primary factor driving patient response to COVID-19 screening hotlines (human or chatbot) were users’ perceptions of the agent’s ability (Dennis et al. 2020, p. 1730). A secondary factor in persuasiveness, satisfaction, likelihood of following the agent’s advice and likelihood of use was the type of agent, with participants reporting that they viewed chatbots more positively in comparison with human agents. This might be both positive and negative from the perspective of professionals. One of the positive aspects is that healthcare organisations struggling to meet user demand for screening services can provide new patient services. However, one of the downsides is patients’ overconfidence in the ability of chatbots, which can undermine confidence in physician evaluations. If health-consulting chatbots are able to evoke feelings of trust among patients, the latter will be more willing to disclose medical information to them and can become more vulnerable to, for example, data hijacking by companies (Pasquale 2020, p. 51).

Based on physician perceptions regarding the use of healthcare chatbots, including their benefits, challenges and risks to patients, Palanica et al. (2019) concluded that the majority of physicians believed that chatbots were unable to effectively care for all patient needs or understand or display human emotion. Because chatbots lack the intelligence to accurately assess patients, they cannot provide detailed clarifications regarding patient assessments, are unable to assess emergency health situations or may indirectly harm patients by not knowing all the personal factors associated with specific patients. In addition, many physicians stated that healthcare chatbots are associated with the risk that patients may self-diagnose too often, that patients may not understand the diagnoses or that patients may not feel adequately connected to their primary physician (Palanica et al. 2019).

Pasquale (2020, p. 57) has reminded us that AI-driven systems, including chatbots, mirror the successes and failures of clinicians. However, machines do not have the human capabilities of prudence and practical wisdom or the flexible, interpretive capacity to correct mistakes and wrong decisions. As a result of self-diagnosis, physicians may have difficulty convincing patients of their potential preliminary, chatbot-derived misdiagnosis. This level of persuasion and negotiation increases the workload of professionals and creates new tensions between patients and physicians. Physicians’ autonomy to diagnose diseases is no end in itself, but patients’ trust in a chatbot about the nature of their disease can impair professionals in their ability to provide appropriate care for patients if they disregard a doctor’s view.

UK health authorities have recommended apps, such as Woebot, for those suffering from depression and anxiety (Jesus 2019). Pasquale (2020, p. 46) pondered, ironically, that cheap mental health apps are a godsend for health systems pressed by austerity cuts, such as Britain’s National Health Service. Unfortunately, according to a study in the journal *Evidence Based Mental Health*, the true clinical value of most apps was ‘impossible to determine’. To develop social bots, designers leverage the abundance of human–human social media conversations that model, analyse and generate utterances through NLP modules. However, the use of therapy chatbots among vulnerable patients with mental health problems bring many sensitive ethical issues to the fore. For instance, Galitsky’s (2019) experiments with chatbot training showed that people and deep learning chatbots lose their train of thought during conversations, make loose associations between topics (tangentially jumping from one topic to another, apparently at random or on the barest of associations) and give answers to unrelated questions.

It is important to emphasise here that chatbots or more advanced AI-driven systems complement rather than replace medical professionals (Powell 2019). The problem, however, is that new technologies and the inconveniences associated with their use, as well as the increasing amount of data provided within them, are in themselves a new burden on health professionals. Of course, some technologies do prove their worth and gradually facilitate many work processes. However, this is not always the case. Powell (2019) used the example of an early study of an algorithm-based triage tool in primary care. His research showed that physicians lacked trust in the ability of the machine to take clinical risks and worried about issues of governance and accountability, such that the sensitivity of the tool, in terms of the urgency of triage, was consistently set at a threshold that would increase urgent clinical workload rather than reduce it. If there is a great amount of uncertainty in the functioning of a system, professionals are unlikely to trust the outcomes and predictions of said system. This leads to ‘cognitive load’ (Zhou et al. 2017), which affects work memory and, thus, delimits cognitive resources, further increasing the level of distrust and misreading of uncertainties. Conversely, if professionals are informed about the possible uncertainties involving the system and its outcomes, they are more likely to trust the system, have lower cognitive load and are able to interpret uncertainties more thoroughly.

While some doctors welcome the emergence of health chatbots, others are more cautious and seek more evidence to separate hope from hype. Pasquale (2020, p. 57) has cautioned that the deployment of chatbots in health care cannot follow a simple market logic; otherwise, cheap apps might quickly become the new gatekeepers of access to specialty care, or they may usurp human doctors in many patient cases. Galitsky (2019) stated that more consistent agreement on the use of healthcare chatbots was apparent with reference to their potential logistical benefits as well as their challenges and potential risks to patients. The many perceived challenges and risks associated with healthcare chatbots would need to be addressed before the technology is widely endorsed by practicing physicians. These challenges may be because of concerns involving regulation and physician remuneration, which supports other relevant research demonstrating that physicians are less likely to use telemedicine services if they are not adequately compensated for their time and effort. Healthcare professionals with specialised knowledge, who provide especially important services, should have the power to organise and control their own work regarding how the implementation of technologies will change their decision-making capabilities.

So far, there has been poor adoption of chatbot technologies among physicians, and patients have shown weak adherence to them (Palanica et al. 2019). According to Palanica et al. (2019), ‘[t]his may be because of the perceived lack of quality or accountability that is characterized by computerized chatbots as opposed to traditional face-to-face interactions with human physicians’. Thus, the patient may feel that they are not as connected to the HCP as in the context of face-to-face interaction. Furthermore, a chatbot can also do harm to a patient if it does not know all the details and personal factors associated with the patient. The physicians did agree that chatbots had potential logistical benefits and that ‘[t]he many perceived challenges and risks associated with health care chatbots would need to be addressed before the technology is widely endorsed by practicing physicians’ (Palanica et al. 2019). Palanica et al. noted that it was still not entirely clear whether chatbots were better overall at improving the various clinical health outcomes of patients and why they should be adopted compared with traditional methods of care, that is, information coming from a human physician. Pasquale (2020, p. 56) called for caution because chatbots (and other ADMs) could lead to ‘a deep social division’, a society divided ‘between those who experience human-to-human connection and those relegated to software and machines’.

Due to the rapid digital leap caused by the Coronavirus pandemic in health care, there are currently no established ethical principles to evaluate healthcare chatbots. Shum et al. (2018, p. 16) defined CPS (conversation-turns per session) as ‘the average number of conversation-turns between the chatbot and the user in a conversational session’. However, these kinds of quantitative methods omitted the complex social, ethical and political issues that chatbots bring with them to health care. As the digitalisation of health care is increasingly becoming all-encompassing, there is a pressing need to develop novel guidelines, frameworks or even rules that ensure that digital healthcare solutions are socially, ecologically and ethically sustainable and do not jeopardise the development of human capabilities.

## Conclusion

In this article, we employed a proactive approach to the potential chatbot breakthrough in health care in the context of the COVID-19 pandemic in order to discuss how the emergence of task-oriented chatbots can influence the nature of clinical practices and the expert-client relationship. One of the fundamental questions was the extent to which chatbots should extend the capabilities of clinicians or replace their capabilities with complex algorithmic systems. Based on the previous research on health chatbots, consultation chatbots have been found to play a beneficial role in supporting, motivating and coaching patients and streamlining organisational tasks. In particular, chatbots could become a surrogate for nonmedical caregivers. In our analysis, the following general points can be provided, although these elements are not yet grounded in evidence-based knowledge:

- In general, chatbots are able to quickly provide patients with information about their health problems based on the symptoms reported on respective platforms. In addition, they can connect patients to HCPs.
- One key aspect is that chatbots are regarded as devoid of human features (in the usual sense). This enables people to see them as more trustworthy and non-judgmental. Thus, people seem to be more willing to share medical information with them—e.g. people use chatbots to diagnose more intimate conditions, such as STDs.
- Chatbots are more efficient in the sense that they can function 24 h per day and do not tire or fall ill.
- It seems that trust in chatbots—when they function as they should—has positive effects on trust in HCPs.

Technological systems in health care are often highly complex in the sense that experts rarely have a full understanding of system design or functionality. When problems are recognised, they can rarely be traced to a single factor because different agents are involved in the configuration of the system. This inability to reliably anticipate the effects of development options undermines the creation of standards for the development of ‘functional’ and ‘ethically and socially sustainable’ chatbots. Therefore, we argue that technologies such as chatbots should be viewed primarily from the perspective of systemic change in order to outline their impact on clinical practices. Considering this systemic perspective in the implementation of chatbots, we found a number of risks and concerns, from the perspective of HCPs, regarding how chatbots may transform clinical capabilities and change patient-clinician relationships in clinical practices in the long run:

1. Negative impacts on the acquisition and use of practical knowledge in clinical work

Rapid diagnoses by chatbots can erode diagnostic practice, which requires practical wisdom and collaboration between different specialists as well as close communication with patients. HCP expertise relies on the intersubjective circulation of knowledge, that is, a pool of dynamic knowledge and the intersubjective criticism of data, knowledge and processes.

(2) The business logic behind technological solutions may start to drive health care

When chatbots are developed by private healthcare companies, they usually follow the market logic, such as profit maximisation, or at the very least, this dimension is dominant. Through the rapid deployment of chatbots, the tech industry may gain a new kind of dominance in health care. AI technologies, especially ML, have increasingly been occupying other industries; thus, these technologies are arguably naturally adapted to the healthcare sector. In most cases, it seems that chatbots have had a positive effect in precisely the same tasks performed in other industries (e.g. customer service).

(3) The risks of incompleteness and the limitations of healthcare chatbots

Due to the limitations and potential incompleteness of healthcare chatbots, they cannot provide a detailed assessment of a patient’s condition. Especially in emergency health situations, they may harm patients as they may not know the full details of the personal factors associated with the patient. Thus, the information that chatbots provide represents very limited expertise. To be more efficient, chatbots need to be up to date on medical regulations, ethical codes and research data. Their limits must be understood and explicitly communicated to patients.

(4) Patients’ responsibility of self-care can increase alongside chatbot development

There are risks involved when patients are expected to self-diagnose, such as a misdiagnosis provided by the chatbot or patients potentially lacking an understanding of the diagnosis. One of the downsides is patients’ overconfidence in the ability of chatbots. If experts lean on the false ideals of chatbot capability, this can also lead to patient overconfidence and, furthermore, ethical problems.

(5) Trust decay

As computerised chatbots are characterised by a lack of human presence, which is the reverse of traditional face-to-face interactions with HCPs, they may increase distrust in healthcare services. HCPs and patients lack trust in the ability of chatbots, which may lead to concerns about their clinical care risks, accountability and an increase in the clinical workload rather than a reduction.

(6) Increased tendency of conflict

As a result of patient self-diagnoses, physicians may have difficulty convincing patients of their potential preliminary misjudgement. This persuasion and negotiation may increase the workload of professionals and create new tensions between patients and physicians.

(7) Chatbots can form unexpected new gateways or bottlenecks in health care

New technologies may form new gatekeepers of access to specialty care or entirely usurp human doctors in many patient cases.

(8) Chatbots can increase workload and costs

Sophisticated AI-based chatbots require a great deal of human resources, for instance, experts of data analytics, whose work also needs to be publicly funded. More simple solutions can lead to new costs and workload when the usage of new technology creates unexpected problems in practice. Thus, new technologies require system-level assessment of their effects in the design and implementation phase.

(9) Negative impact on employee well-being and motivation at work

The implementation of chatbots without the consent of HCPs may reduce HCP motivation and capabilities to control their own work. In the long run, clinical practices can become monotonous: the shift from operator to supervisor, which also decreases the embodied and emotional relationship between HCPs and patients.

(10) The possibility of pushing health care into a domino effect

Like falling dominoes, the large-scale deployment of chatbots can push HCPs and patients into novel forms of healthcare delivery, which can affect patients’ access to care and drive some to new provider options. Due to partly automated systems, patient frustration can reach boiling point when patients feel that they must first communicate with chatbots before they can schedule an appointment. The dominos fall when chatbots push patients from traditional clinical face-to-face practice to more complicated automated systems.

We acknowledge the difficulty in identifying the nature of systemic change and looking at its complex network-like structure in the functioning of health organisations. Nonetheless, we consider it important to raise this point when talking about chatbots and their potential breakthrough in health care. We suggest that new ethico-political approaches are required in professional ethics because chatbots can become entangled with clinical practices in complex ways. It is difficult to assess the legitimacy of particular applications and their underlying business interests using concepts drawn from universal AI ethics or traditional professional ethics inherited from bioethics. Ethical issues within chatbots in health professional work do not simply fall in the domain of individual or organisational challenges; they require policy decisions on how the new tools can be implemented in order to automate decisions based on human assessment. Insufficient consideration regarding the implementation of chatbots in health care can lead to poor professional practices, creating long-term side effects and harm for professionals and their patients. While we acknowledge that the benefits of chatbots can be broad, whether they outweigh the potential risks to both patients and physicians has yet to be seen.

## Data Availability

Not applicable.

## Footnotes

1 https://koronavilkku.fi/en/
